# Special Issue “Molecular Progression of Genetics in Breeding of Farm Animals”

**DOI:** 10.3390/ijms27209006

**Published:** 2026-10-11

**Authors:** Karel Novák

**Affiliations:** Department of Genetics, Genomics and Livestock Breeding, Institute of Animal Science, Přátelství 815/109, 104 00 Prague, Czech Republic; novak.karel@vuzv.cz

## 1. General Points

This Special Issue, “Molecular Progression of Genetics in Breeding of Farm Animals”, aims to map current trends at the intersection of molecular genetics/biology and livestock breeding, as well as to illustrate these trends through selected examples of applied molecular approaches. This Special Issue covers breeding for health traits based on genetic and genomic information, the study of which can provide potential solutions for sustainable breeding programmes. Consequently, this Special Issue sought to summarise the use of variability in genes responsible for health traits in relation to the current state of applied genome sequencing in livestock species.

This subject area is difficult to comprehensively cover within a single issue, but further knowledge of the molecular determination of individual traits and their application to breeding can create a general outline of this research direction.

## 2. Polymorphism in Innate Immune Genes as a Resource for Breeding

Improvements in the innate immune system through the inclusion of causal genes in selection schemes are based on good general knowledge of both the function and diversity of innate immune genes in livestock species; however, further investigation is still needed, as emphasised by Novák and Samaké in their review article included in this Special Issue (contribution 1). Furthermore, it appears that the other members of the Toll signalling pathway—in addition to the Toll-like receptors themselves—have not yet been utilised to their full extent as breeding targets aimed at improving health status and disease resistance.

This contrasts with the frequently described links of these genes to health disorders and hereditary predispositions in human medicine (contribution 1). Regarding the Toll signalling pathway adaptor MyD88, which is described in detail in the cited review, the number of publications on the effects of its diversity in biomedicine, reaching 1500 according to the Web of Science database, exceeds the number of analogous articles dealing with livestock species by approximately one hundred times.

Consequently, this difference indicates a knowledge gap regarding the role of diversity in this adaptor for the formation of health traits in economically important animals. An analogous difference can also be observed in other adaptors of the Toll-like pathway.

## 3. Surveying Genetic Variability as a Resource for Resilience Breeding

Livestock breeders are focusing their attention on genes that control the resilience of farm animals. Breeding for this ability can be considered a prospective direction, and this area is becoming increasingly relevant due to climate change and growing demands for animal welfare. In a wider sense, the resilience trait is interpreted as an animal’s ability to manage disturbances in their environment and to quickly restore normal functioning, as defined by Laghouaouta et al. (contribution 2). Since climate change, beyond any doubt, complicates the housing of the traditional breeds of animals in temperate regions, resilience against temperature shocks is often interpreted as general resilience. Being resilient to high temperatures is an important precondition for the increase in highly efficient modern breeds in tropical and subtropical regions. The trend of using traditional breeds from these regions is a consequence of insufficient heat resistance management in modern commercial breeds, as documented by Van Marle-Köster and Visser [1]. Regarding intentional breeding for resilience to environmental factors, this type of programme has only started relatively recently because breeding for utilitarian traits has always been prioritised. However, it must also be recognised that breeding aimed at climate adaptation and stable phenotypes occurred spontaneously as a part of the historical dissemination of main livestock species into new regions.

This is exemplified by the spread of cattle into Africa through the Suez area, approximately 7000 years ago [2], and further across the tropical belt to the coast of the Gulf of Guinea. This process resulted in a considerably better adaptation of the historical local breeds of cattle to the meteorological factors than of the imported, albeit otherwise highly productive, modern cattle breeds [3].

A similar situation can be observed among the historical and highly productive modern breeds of other main livestock species [4]. Researchers expect that the application of new approaches of molecular genetics and genomics will allow for the identification of key determinants of resilience and their transfer through hybridisation, aiming to improve the main production breeds.

## 4. Studying Genomes of Livestock Species Backwards to Understand Modern Populations

Genomic and archaeogenetic methods allow us to identify key determinants for interpreting the diversity observed in the current populations of livestock species, including modern breeds that are subject to intensive breeding.

Similarly, examining the consequences of long-term natural selection for limiting traits allows us to identify the genes under selection and to specify their role [5]. Zhong et al. used this approach (contribution 3), which is independent of setting experiments or performing association studies in extant populations, to identify the genes or regions under long-term selection by comparing a set of cattle breeds adapted to different conditions. Both copy-number variation in the presumed relevant genes and historically established selection signatures identified pathways related to a group of key genes for heat tolerance, disease resistance, fat metabolism, and environmental adaptation. Candidate genes associated with reproduction and the development of sperm and oocytes were identified as well. Although the study published within this Special Issue is primarily aimed at improvements in the breeding of Chinese Hainan yellow cattle, both the approach used and the key findings are generalisable to cattle breeding.

## 5. Decoding the Role of Key Genes in Association Studies

Association studies performed as a prerequisite for applications in livestock species breeding can provide necessary information about the functions performed by the variable genes. This approach, as carried out by Wang et al. (contribution 4), elucidates the role of genes and enzymes governing base excision repair in newborn piglets. Representative *MUC2* gene expression in the duodenum and jejunum is known to prevent intestinal disorders in piglets during the weaning stage [6], as this gene produces mucin—a highly glycosylated protein that forms a protective barrier for intestinal epithelial cells. The role of this animal’s genotype is exemplified by the difference between the Lin and Landrace pig breeds. The results of this study notably enhance our understanding of *MUC2*, but its role in specific mucosal malignancies and mucinous adenocarcinomas of the lung or breast is not yet clear [7].

Further research in this direction can uncover additional functions of this group of genes for base excision repair, which have only been partially documented.

## 6. Applications in the Breeding of Wool-Producing Species

Economically important traits considered for molecular breeding also include the quality of hair collected from sheep and goat breeds specifically reared for this purpose. Though the progress in synthetic fibre use has had strong economic impacts, high-quality wool from traditional breeds cannot be replaced. This is illustrated by Zhu et al. (contribution 5) in the Special Issue, as they examined a new role of the specialised transcription factor Csdc2. This protein has been found to regulate secondary hair follicles in cashmere goats in a previous study [8]. Generally, the transcription factor Csdc2 (cold shock domain-containing protein C2), also known as the RNA-binding protein PIPPin, encodes an RNA-binding protein with predicted binding activity to the 3′-UTR of mRNA. In this way, the Csdc2 protein regulates mRNA stability and thereby exerts post-transcriptional control [9]. The protein is localised in both the nucleus and cytoplasm, more specifically in the nucleoplasm and cytosol.

Another study (contribution 5) has demonstrated that the *CSDC2* gene is expressed in the hair bulb of secondary hair follicles and regulates at least 25 genes, including the roundabout guidance 2 (Robo2) receptor. Accordingly, knocking down the *CSDC2* gene in cell culture results in a reduced expression of key genes involved in hair follicle development.

In addition to strategies for molecular breeding for cashmere production in goats, this research overlaps with that in a number of species aimed at explaining the differentiation processes based on a similar mechanism or even the direct involvement of Csdc2 [9]. This assumption is also supported by an extremely high number of 6117 binding motifs across the goat genome and their pronounced evolutionary conservation (contribution 5) [10].

## 7. Genomic Possibilities in Breeding for Poultry Egg Quality

Approaches based on ‘-omics’ are useful in reducing the decline in quality of eggs with age in laying hens. These approaches are highly efficient tools that make this task practical and achievable. An association study conducted by Gao et al. (contribution 6), based on both genomic and transcriptomic data, identified as many as 77 genes associated with albumen quality, as well as 259 genes correlating with this trait. However, only four genes can be considered robust regulators. Although there has been no additional independent evidence to date, it is not surprising that these regulatory genes act via mitochondrial function, antioxidant defence, and membrane transport. Despite their primary function of reducing the impact of age on the production of chicken eggs in commercial flocks, these results can apply to general biology.

This again indicates the potential use of livestock species as models for biomedical or general biochemical research, as it has been known that livestock association studies can complement research in these fields [11]. Not all studies can be accomplished using rodent models, so the defined genetic background in livestock species, standardised environmental factors, and abundant populations used for analysis facilitate the experiment design and increase the resolution power of the statistical methods applied to functional population genetics. Therefore, interconnected studies of analogous traits carried out in standardised livestock populations and biomedical projects in patient cohorts are fully justified and can provide important data. This type of analysis has already been recommended for publications regarding the complete pig genome [12], and this particular goal was also defined in extensive projects on livestock genomics, such as the Functional Annotation of Animal Genomes project [13].

## 8. Genetic Factors Controlling Oxidative Stress

Bone fragility is a harmful trait in many livestock species and can become a limiting factor for production. Even more importantly, it is an indicator trait reflecting the equilibrium of genetic and nutritional factors controlling a set of physiological parameters. These parameters include not only the causal processes of bone fragility, such as osteopenia and osteoporosis, but also the oxidative processes in unsaturated lipids in the organism [14].

The genetic basis of resistance to oxidative stress is examined by Kowalczyk et al. (contribution 7). Adverse consequences of oxidative stress can be diminished in newborn piglets through a specific diet consisting of selected unsaturated fatty acids. The diet is aimed at increasing the activity of base excision repair (BER) enzymes, which play a protective role in preventing oxidative damage, such as degradative changes to DNA, among other effects. Adhering to a designed diet containing oils rich in long-chain n-3 PUFAs (polyunsaturated fatty acids) during pregnancy can significantly decrease the activity of BER enzymes. Correspondingly, mRNA expression of BER genes (TDG, MPG, and OGG1) was observed in piglets from gilts; notably, natural oil forms appeared to be more effective than their nanoparticle formulations.

This research opens up new avenues to treat disruptions in fatty acid metabolism, including the subsequent steps leading to arachidonate products, which directly affect a set of important traits relating to bone formation and health. From the perspective of agricultural production, bone fragility is one of the limiting factors in large-scale pig production worldwide [15], and similarly, bone damage is a limiting factor for intensive poultry production [16]. This situation is often attributed to the collateral effects of the previous selection aimed at the intensification of animal production, expressed in terms of quantitative parameters of growth or clutch size [17]. Nevertheless, targeted research on the genetic factors that affect the expression of bone fragility in livestock species might play a crucial role in overcoming this undesirable and currently limiting consequence of intensive animal husbandry.

## 9. Conclusions

The overlap between the breeding of livestock species and the current rapid progress in molecular genetics and genomics allows us to examine progress in many research areas. Representative works documenting these approaches have been collected in this Special Issue in order to support further development.

## Abbreviations

The following abbreviations are used in this manuscript:

3′-UTR	3′-untranslated region of mRNA
BER	Base excision repair enzymes
Csdc2	Cold shock domain-containing protein C2
*MUC2*	Mucin 2 encoding gene
n-3 PUFAs	Omega-3 polyunsaturated fatty acids
Robo2	Roundabout guidance 2 receptor
